# Is Cold Atmospheric Plasma Selective for Breast Tumor Cells? A Systematic Review

**DOI:** 10.3390/ijms27041710

**Published:** 2026-02-10

**Authors:** Inês Pinheiro, Catarina Almeida-Ferreira, Carlos Miguel Marto, Francisca Rodrigues, Francisco Caramelo, Maria Filomena Botelho, Mafalda Laranjo

**Affiliations:** 1 Coimbra Institute for Clinical and Biomedical Research (iCBR) Area of Environment Genetics and Oncobiology (CIMAGO), Institute of Biophysics, Faculty of Medicine, University of Coimbra, 3000-548 Coimbra, Portugalcatarinalmeidaferreira@gmail.com (C.A.-F.); cmiguel.marto@uc.pt (C.M.M.); anafpcrodrigues@gmail.com (F.R.); fcaramelo@fmed.uc.pt (F.C.);; 2 Center for Innovative Biomedicine and Biotechnology (CIBB), University of Coimbra, 3000-548 Coimbra, Portugal; 3 Faculty of Pharmacy, University of Coimbra, 3000-548 Coimbra, Portugal; 4 Clinical Academic Center of Coimbra (CACC), 3004-561 Coimbra, Portugal; 5 Institute of Integrated Clinical Practice and Laboratory for Evidence-Based Sciences and Precision Dentistry, University of Coimbra, 3000-075 Coimbra, Portugal; 6 Institute of Experimental Pathology, Faculty of Medicine, University of Coimbra, 3000-548 Coimbra, Portugal; 7 Centre for Mechanical Engineering, Materials and Processes (CEMMPRE), Advanced Production and Intelligent Systems (ARISE), University of Coimbra, 3030-788 Coimbra, Portugal

**Keywords:** cold atmospheric plasma, breast cancer cells, nonmalignant cells, selectivity, systematic review

## Abstract

Breast cancer (BC) is the most diagnosed cancer among women and ranks as the fourth leading cause of cancer-related deaths worldwide. Current main treatments have significant issues, including a lack of selectivity for tumor cells. Over the past decade, cold atmospheric plasma (CAP) has been examined as possible therapy for cancer. Therefore, this systematic review aimed to understand if there is selectivity of CAP or plasma-activated solutions (PAS) for BC cell lines in vitro. The research in PubMed, Embase, Web of Science, and Cochrane databases resulted in 243 articles, and of these, 32 in vitro studies were included. MDA-MB-231 and MCF-10A cells were the most used. MTT, MTS, SRB, trypan blue, clonogenic, resazurin, luciferin, annexin-V/propidium iodide, reactive oxygen species (ROS), and scratch assays were carried out. This research showed that CAP and PAS tended to reduce the viability of cancer cells, causing less damage to nonmalignant cells, demonstrating selective or partial selectivity toward cancer cells. One of the mechanisms potentially underlying this selectivity is the elevated ROS basal levels typically found in cancer cells. These high ROS levels may lead to increased expression of membrane aquaporins and a reduced capacity for antioxidant defense, contributing to heightened membrane permeability and vulnerability to oxidative damage. Additionally, the treatments also tended to reduce the migration of BC cells. CAP treatment enhanced several other therapies’ effectiveness. However, the differences in experimental protocols, treatment approaches, equipment features, and exposure times observed across the studies made it impossible to carry out the planned meta-analysis. Existing in vitro evidence indicates that CAP/PAS exhibit partial selectivity for breast cancer cells, but due to the heterogeneity in experimental protocols, the consistency of selectivity remains to be verified. Further research is needed to elucidate their mechanisms of action and to standardize experimental methods.

## 1. Introduction

Cancer is a multistep process in which normal cells gradually turn into malignant cells due to successive mutations [1]. This process is influenced by a combination of genetic predisposition, lifestyle, and environmental exposure factors. The latter is increasingly recognized as an important risk factor for cancer and the global incidence of the disease [2]. During carcinogenesis, cancer cells acquire abilities, such as uncontrolled proliferation, invasion, and metastatic potential, which are designated as hallmarks of cancer [3]. According to statistics from the World Health Organization (WHO), breast cancer (BC) was the most prevalent cancer among women in 2022, with around 2.3 million new cases [3]. It was also ranked as the fourth major cause of cancer-related mortality worldwide in 2022, with 666 103 deaths [4]. This neoplasm can be categorized molecularly based on the expression of hormone receptors, specifically estrogen receptors (ER), progesterone receptors (PR) and human epidermal growth factor receptor type 2 (HER2) [5]. The three intrinsic molecular subtypes are hormone receptor-dependent (subdivided into luminal A and B), HER2-enriched, and triple-negative breast cancer (TNBC), which expresses none of the receptors [6]. Such classification allows disease categorization and treatment selection. Also, the Tumor-Node-Metastasis (TNM) classification is used to accurately stage BC, guide treatment choices, track therapy response, and adjust treatment regimens [7]. This standardized method evaluates the primary tumor’s size and relationship with surrounding structures, regional lymph nodes’ characteristics, and the presence or absence of distant metastasis, reflecting the tumor’s prognosis [8]. Currently, the main treatment options for BC are surgery, chemotherapy, radiotherapy, and targeted therapies [9]. However, they present limitations, including a lack of specificity in treatment, development of drug resistance, high recurrence rate due to the difficulty in eradicating cancer stem cells, and associated side effects [9,10]. Thus, advancing research into new therapeutic approaches is crucial. Plasma, considered the fourth state of matter, has been described as an excellent electrical conductor with enough energy to positively and negatively ionize charged particles [11]. It is classified based on the temperature of the electrons compared to other particles into two types: thermal and nonthermal [12]. Cold atmospheric plasma (CAP), also known as nonthermal plasma or low-temperature plasma, is characterized by a state in which the electrons are at a different temperature from the heavier particles, which remain at room temperature [12,13]. CAP can be generated using a range of gases like helium, argon, and air through techniques such as atmospheric plasma jet, plasma needle, and dielectric barrier discharge (DBD). In DBD, an electrical discharge occurs between two electrodes separated by a dielectric barrier, resulting in the ionization of the gas between them [14]. CAP has a wide array of applications in biomedicine, including dentistry, tissue regeneration, and dermatology. Recently, it has been explored as a potential anticancer treatment [15]. Several studies have shown that CAP significantly reduced cancer cell proliferation and viability in vitro, as well as reduced tumor size in vivo, without side effects, in several types of cancer, for instance, BC [16,17], retinoblastoma [18], prostate cancer [19], glioblastoma [20], lung cancer [21], leukemia [22], and pancreatic cancer [23]. Despite the demonstrated efficacy, the CAP mechanism remains unclear; however, there are some hypotheses, including the direct effects of ultraviolet (UV) radiation on cells through absorption of the radiation and alteration of cellular biomolecules, the impact of electromagnetic fields on cellular calcium permeability, deoxyribonucleic acid (DNA) integrity, and mitosis, and the production of reactive oxygen and nitrogen species (RONS) that alter the intracellular redox balance [24,25]. RONS are recognized as key mediators of cellular responses to CAP treatment. Moreover, selectivity has been mentioned as a relevant feature of CAP treatment. The investigations have shown that CAP is more cytotoxic for tumor cells than nonmalignant cells, showing promising results in various tissues [17,26,27,28,29]. The CAP exposure of keratinocytes and fibroblasts for durations of less than 2 min was not associated with a significant increase in cellular toxicity or apoptosis [30]. Similarly, the metabolic activity of pancreatic cancer cells is reduced compared to primary murine fibroblast cells. Apoptosis increased in CAP-treated pancreatic cancer cells, opposing nonmalignant cells [31]. Concerning plasma-activated solution (PAS) treatment, normal fibroblasts showed an increase in nuclear factor erythroid 2-related factor 2 (NRF2) protein levels, in contrast to human fibrosarcoma cells. Moreover, it was observed that NRF2 translocated to the nucleus in PAS-treated fibroblasts, enhancing the expression of antioxidant proteins [32]. Both CAP and PAS treatments exhibited selectivity towards bone cancer cells, reducing their cellular viability to 30% while preserving nonmalignant cell lines [33]. While the specific mechanisms underlying this apparent selective impact of CAP and PAS on cancer cells remain unclear, it may be attributed to the inherently elevated baseline RONS levels in cancer. Treatment-induced RONS elevation can overcome the apoptotic threshold in malignant cells, whereas in non-malignant cells, their lower baseline RONS levels and antioxidant defenses are maintained, effectively mitigating oxidative stress [34]. Based on all this evidence, we hypothesized that CAP and PAS selectively induce cytotoxicity in BC cells. Accordingly, the main goal of this systematic review was to determine whether CAP and PAS are more selective in BC cells than in nonmalignant cells.

### Mechanisms of Action of CAP

CAP has been shown to mediate its antitumor effects through the combined action of chemical and physical factors that explain its selective killing effect on tumor cells [12]. Among these factors, RONS are widely recognized as the principal mediators of the biochemical and molecular effects induced by CAP [35].

Tumor cells are known to exhibit mitochondrial dysfunction and altered metabolism, leading to greater basal levels of intracellular RONS than nonmalignant cells [9,36]. During CAP exposure, plasma-generated RONS can penetrate the cell membrane either through aquaporin channels or membrane destabilization with pore formation induced by lipid peroxidation [35,37,38]. The low cholesterol content in tumor cell membranes, for example, makes these membranes more permeable to RONS [39]. Once internalized, excessive RONS disrupt mitochondrial integrity, induce loss of membrane potential, and induce oxidative damage in lipids and DNA, including double-strand breaks [40,41]. Calcium plays a key role in cell signaling, affecting survival pathways [42]. It is thought that the increase in cytoplasmic calcium concentration associated with CAP treatment causes an overload of mitochondria, thereby inducing apoptosis via the intrinsic pathway through cytochrome c release [30,43]. The convergence of extracellular RONS delivered by CAP with enhanced endogenous RONS production overwhelms the antioxidant capacity of tumor cells, leading to apoptosis [44].

**Figure 1:**
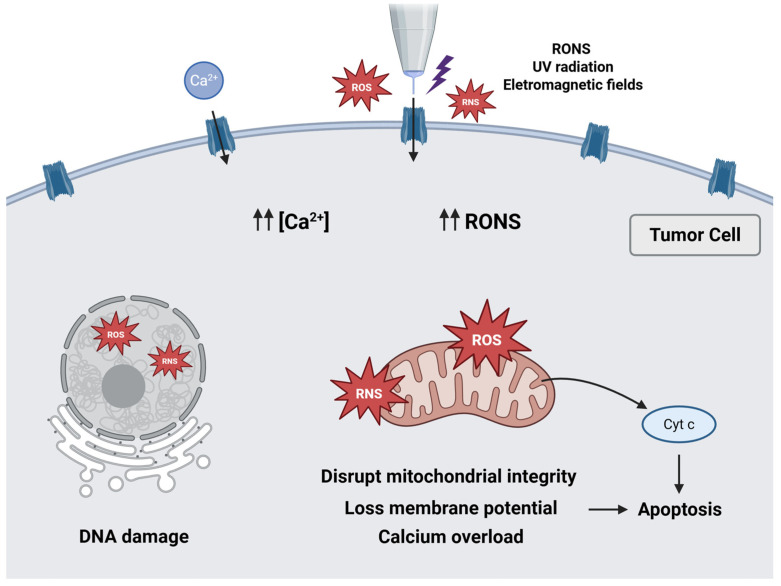
Schematic illustration of the mechanisms of action underlying the antitumor effects of CAP. Created with Biorender.com.

Besides these chemical components, UV radiation generated during plasma exposure can directly damage DNA, proteins, and lipids, while simultaneously promoting intracellular RONS formation. Moreover, electric fields have been proposed to modulate cell membrane permeability, reorganize cytoskeletal structures, and enhance calcium influx through voltage-dependent channels, thereby amplifying pro-apoptotic signaling cascades [17,25]. Figure 1 summarizes the main mechanisms of action proposed.

Recently, ozone has been proposed as a new effector molecule that preferentially triggers tumor cell apoptosis as a result of the high metabolic activity of these cells, mostly leaving the nonmalignant cells unaffected [45].

## 2. Methods

The systematic review followed the Preferred Reporting Items for Systematic Reviews and Meta-Analyses (PRISMA) guidelines [46]. The protocol was registered in the Open Science Framework (OSF) with the registration code 10.17605/OSF.IO/VRZP3. The research question was structured using the population, intervention, comparison, and outcome (PICO) methodology: Is cold atmospheric plasma more selective for BC cells than for nonmalignant cells? (Table 1).

In addition to the primary outcome, other parameters were also considered, including cell death, protein and gene expression, cell migration, reactive oxygen species (ROS) levels, proliferation, glucose and lactate concentrations, adenosine triphosphate (ATP) levels, cellular metabolism, colony formation, as well as antioxidant defenses detection.

The practical definition of selectivity was defined as a higher cytotoxic effect of CAP or PAS on BC cells, accompanied by a less reduction or absent effect on nonmalignant cells, when both were assessed under the same experimental conditions within the same study. Given the heterogeneity of assays, endpoints, and treatment protocols, selectivity was qualitatively interpreted based on differences in cell viability, proliferation, or apoptosis, for example. Studies were accordingly classified as selective or partially selective.

### 2.1. Literature Search

The literature search was performed in four databases: Embase, Medline (through PubMed), Web of Science (all databases), and Cochrane Library. A search strategy was constructed for Medline using a combination of MeSH terms and keywords and adapted for the remaining databases. The detailed searches are presented in Supplementary Materials S1. No restriction on publication date was applied, and filters were used to select studies published in English, French, Portuguese, and Spanish. The search was conducted on 18 June 2024 and repeated on 7 March 2025. A manual search was also conducted in the reference list of included studies to find potential additional papers. The search results were imported into Rayyan© website (Qatar Foundation) and duplicates were removed [47].

### 2.2. Eligibility Criteria

Two independent reviewers assessed the eligibility of studies for inclusion, first based on the title and abstract and later on the full text. During the selection process, only in vitro studies were considered, respecting specific inclusion criteria: (1) use of BC and nonmalignant cells, (2) application of CAP alone or in combination with other treatments, (3) studies describing biologic effects of CAP and (4) publication of original results. Studies were excluded if they did not satisfy the following criteria: (1) other types of cancer than BC, (2) no effect of CAP, (3) lack of BC comparisons with nonmalignant cells, and (4) in vivo, in silico, and clinical studies.

### 2.3. Data Extraction

After selecting the studies, detailed information was extracted, incorporating: (1) authors and year of publication, (2) type of BC cell line and nonmalignant cells used, (3) CAP or PAS therapy (i.e., plasma source, equipment parameters such as voltage, gas flow, and exposure times), (4) methodologies applied to both types of cell lines, and (5) the main results obtained from the research.

### 2.4. Risk of Bias Evaluation

The risk of bias in the included in vitro studies was assessed using the Toxicological Data Reliability Assessment Tool (ToxRTool), which offers a standardized framework for evaluating data consistency and quality [48]. The quality assessment process for the eligible studies incorporated in this systematic review was independently conducted by two authors.

## 3. Results

### 3.1. Study Selection

**Figure 2:**
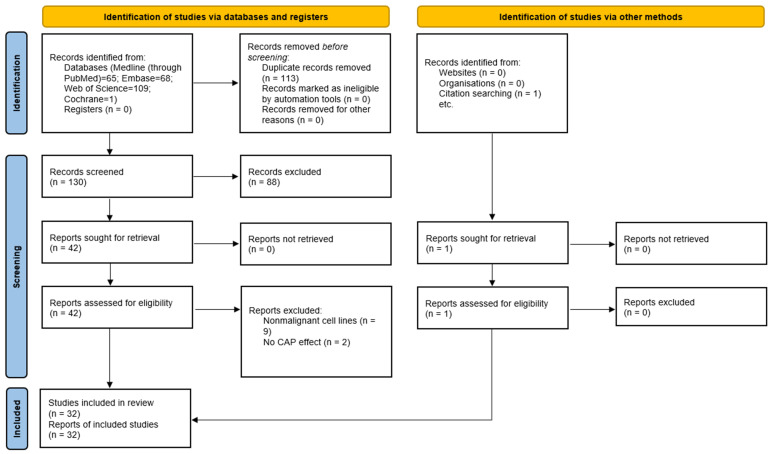
PRISMA flow diagram for selecting studies in this systematic review.

The search initially identified a total of 243 studies: 68 from Embase, 65 from PubMed, 109 from Web of Science, and one from the Cochrane Library. After the removal of 113 duplicate articles, 130 articles were retained for subsequent evaluation. During the screening by title and abstract, 88 studies were excluded, leaving 42 articles for a detailed review of the full text. Among these, nine articles were excluded due to the lack of comparisons with nonmalignant cells, and two articles were excluded because they did not evaluate the efficacy of CAP. Finally, 32 articles published between 2013 and 2025 were included in the systematic review. Figure 2 shows the PRISMA flow diagram summarizing the process of selecting studies for this systematic review.

### 3.2. Characteristics of Studies

Table 2 presents the main characteristics and results of the 32 included in vitro studies. The studies explored two CAP irradiation approaches, direct irradiation (CAP) and indirect methods involving PAS, consisting of previously exposed solutions. CAP or PAS, combined with other therapies, was also included. Table 3 summarizes the percentage reduction in cell viability caused by the different plasma treatment protocols, highlighting the quantification of the BC and nonmalignant cell lines.

#### 3.2.1. CAP Treatment

The cell lines MCF-10A, with a normal phenotype, and MDA-MB-231, representative of TNBC, stood out as the most used. Other nonmalignant cells used were mesenchymal stem cells, human mammary epithelial cells, human keratinocytes, HBL, and fibroblasts. BC cells included AMJ-13, MCF7, AMN3, T47D, SK-BR3, and MDA-MB-453. As shown in Table 2, the MTT [3-(4,5-dimethylthiazol-2-yl)-2,5-diphenyl-2H-tetrazolium bromide], MTS [3-(4,5 dimethylthiazol-2-yl)-5-(3-carboxymethoxyphenyl)-2-(4-sulfophenyl)-2H-tetrazolium], resazurin, clonogenic, cell-injury, scratch assays, ROS quantification and antioxidant defense detection, apoptosis, proliferation, quantitative polymerase chain reaction (qPCR), trypan blue, annexin-V/propidium iodide (PI) for flow cytometry (FC), live-cell imaging, and fluorescence microscopy were performed to investigate the effects of CAP.

The effects of CAP varied depending on the duration of the exposure times, where most treatment protocols exposed cultures to short, up to 120 s of CAP, and a few used longer periods. Adil and his collaborators reported 5–10 s of exposure to CAP causing cytotoxicity of 61.7%, 58.07%, and 68% in the MCF7, AMN3, and AMJ13 cells, respectively, in comparison with nonmalignant breast epithelial HBL cells that remained unaffected [49]. CAP decreased the metabolic activity of MDA-MB-231 by 50% after 30 s exposure, while for keratinocytes (HaCaT), 120 s were required to achieve the same reduction [50]. However, HFF1 fibroblasts had a lower IT50, 61.5 ± 2.1 s, than HCC1806, 69.4 ± 9.3 s, and MCF7, 65.6 ± 7.9 s [17]. Regarding apoptosis, short exposures of 30 s increased its rate in MDA-MB-231 and MCF7, while preserving the viability of nonmalignant cells, MCF-10A [51]. Also, treatment for 60 and 120 s induced apoptosis in the same TNBC cell line (MDA-MB-231) compared to minimal changes in human epithelial MCF-10A cells [52]. Conversely, identical CAP exposure times (60s, in different culture media) showed contradictory apoptosis results with a negative impact on both MCF7, MDA-MB-231, and MCF-10A cell lines [53].

Oxidative stress is a key mechanism underlying CAP action. CAP induces persistent oxidative stress in tumor cells, leading to decreased levels of total reduced glutathione (GSH) and increased levels of oxidized GSH. However, in keratinocytes, the initial oxidative stress is followed by antioxidant recovery [50].

**Table 2:** Characteristics of the included studies.

Author (Year)	Cells		Treatment	Methods	Main Results
	BC	Nonmalignant			(1) Cytotoxic effects of CAP on BC vs. nonmalignant cells (2) Other cellular effects of CAP in both cells (3) Other therapies combined with CAP
**Adil et al. (2020)** [49]	AMJ-13 MCF-7 AMN3	HBL	CAP. Plasma device: 13.2 kV, 70 kHz. Exposure times: 5, 10, 15 s Incubation periods: 3, 24, 48 and 72 h	MTT assay ROS quantification (DCFDA dye)	(1) Brief CAP exposures and incubation periods determined 61.7%, 68%, and 58.07% maximum cytotoxicity on MCF7, AMJ13 and AMN3 cells. HBL cells showed little or no cytotoxicity. (2) The cancer cell lines exhibited the highest levels of ROS, while the HBL showed the lowest levels (*p* < 0.0001).
**Almeida-Ferreira et al. (2021)** [17]	MCF-7 HCC1806	HFF-1	CAP. Plasma device: 60 MHz. Air plasma jet using a sterilized needle with a radius of 0.9 mm and a length of 40 mm. Exposure times: 15, 30, 60, 90, 120 s Incubation: 24 h	SRB assay MTT assay	(1) MCF7 needed 22.4 ± 4.7 s of treatment for a 50% decrease in viability (IT50), while in HCC1806 it required 49.8 ± 10.0 s. For HFF1, IT50 was not computable. (2) The reduction in metabolic activity by 50% was low in HFF1, 61.5 ± 2.1 s, and in HCC1806 and MCF7 it was higher (69.4 ± 9.3 and 65.6 ± 7.9 s, respectively).
**Bekeschus et al. (2020)** [50]	MDA-MB-231	HaCaT	CAP. Plasma device: 1 MHz. Ar plasma jet, kINPen, with 2 L/min flow rate. Exposure time: 30, 60, 120 s Incubation: 6 or 24 h	Resazurin assay GSH assay	(1) 30 s of exposure reduced metabolic activity by 50% in MDA-MB-231, while in HaCaT it needed 120 s to achieve the same reduction. (2) In tumor cells, total GSH levels decreased, and oxidized GSH levels increased consistently. In HaCaT cells, GSH levels dropped in the first 30 s and oxidized GSH increased between 30 and 60 s. These results mean that MDA-MB-231 suffered continuous oxidative stress compared to HaCaT, which showed antioxidant recovery after the initial stress.
**Cheng et al. (2017)** [54]	MDA-MB-231	WTDF	CAP (with or without SMF): direct and indirect treatment. Plasma device: 3.16 kV. He plasma jet with a flow rate of 4.7 L/min. Solution: DMEM Exposure time: 30 s Incubation: 72 h	MTT assay	(1) CAP reduced viability in WTDF by 15% and in MDA-MB-231 by 60%. The SMF in MDA-MB-231 further increased cell death by 25% (direct) and 20% (indirect), with *p* = 0.0379 between direct and indirect treatment.
**Dai et al. (2022)** [55]	SUM159PT SUM149PT MDA-MB-231 MCF-7 SKBR3	MCF-10A	CAP, PAS, and CAP with Atorvastatin. Plasma device: 1.1 kV, 8.8 kHz. He gas flow rate to 1 L/min. Solution: Medium Exposure times: 1, 2, 3, 4, 5 and 10 min Incubation: 8, 16 and 24 h	Viability assay (Cell Counting Kit) Annexin V/PI (FC) ROS quantification (CellROX^®^ Green Reagent) Scratch assay Seahorse assay (ATP generation)	(1) CAP for 2–5 min selectively reduced the viability of SUM159PT, SUM149PT, and MDA-MB-231, with MCF-10A and MCF7 showing slight increases, and SKBR3 remaining unaffected. PAM also doubled apoptosis in the first three, with no effect on MCF-10A, MCF7, and SKBR3. (2) PAS reduced the migration of SUM159PT, SUM149PT, MDA-MB-231, SKBR3 and MCF7 cells, with no effect on MCF-10A. TNBC cells (MDA-MB-231, SUM159PT, SUM149PT) exhibited higher levels of ROS, glycolysis, and ATP production in response to treatment, while MCF-10A had more glycolysis and ATP, but did not respond to PAS. (3) The combination of CAP and atorvastatin significantly increased anticancer efficacy compared to the use of CAP or atorvastatin *per se*, positively affecting cellular viability, apoptosis, and cell migration.
**Fadhil et al. (2024)** [56]	MCF-7 AMJ13	ASCs	PAS. Plasma device: 18–20 kV. Ar gas flow rate 3 L/min. Solutions: RPMI-1640 and MEM Exposure time: 10, 15, 20, and 25 min Incubation: 24 and 48 h	Glucose assay (Cobas Integra Glucose HK Gen. 3 system for quantitative measurement of glucose) Lactate assay (Lactate Dehydrogenase acc. IFCC reagent system)	(2) Exposure increased glucose concentration in MCF7 and AMJ13 after 24 and 48 h (*p* = 0.0006 and *p* = 0.0009; *p* < 0.0001 in both intervals, respectively), while in ASCs this increase was dependent on exposure time (*p* = 0.002 to *p* = 0.0008). Comparing PAS-treated and untreated cells, the treatment with PAS caused a higher increase in glucose uptake in MCF7 and AMJ13 (*p* = 0.016 and *p* = 0.002; *p* = 0.006 and *p* = 0.017, respectively), with lower concentrations in ASCs compared to untreated cells (*p* = 0.02 and *p* = 0.017). LDH activity was higher in AMJ13, indicating a higher metabolic rate.
**Jalili et al. (2016)** [57]	MCF-7	Human fibroblasts	CAP combined with iron oxide (Fe_2_O_3_) NPs. The plasma device consisted of an insulator material through which an inert gas, such as helium, was injected to transport a reactive gas. Exposure time: 5, 15, 30, 45 s Incubation: 24 h	MTT assay	(3) In MCF7, treatments with CAP (30 s and 45 s) and iron NPs significantly reduced the viability (*p* < 0.05; *p* < 0.001 and *p* < 0.05, respectively). The effect was even higher when combining the methods (*p* < 0.001). Human fibroblasts were not significantly affected by any treatment.
**Jezeh et al. (2020)** [58]	MDA-MB-231	Human fibroblasts	CAP and PAS Plasma device: 25 kHz, He gas with a flow of 4 SLPM and O_2_ flow of 20 sccm. Solution: Medium Types of treatment: He (5 kV) and He + 0.5% O_2_ (6 kV) Exposure times to CAP: 1, 2, 3, 4, 5 min Exposure time of PAS to CAP: 1, 2, 3, 4, 5 min Incubation: 48 h	MTT assay	(1) CAP reduced the viability of MDA-MB-231 to less than 40% after 48 h; meanwhile, in fibroblasts, He and He + 0.5% O_2_ reduced viability by 25% and 30%, respectively, after 5 min. With PAS, the reduction was lower, reaching 17% for both gas compositions after 5 min.
**Kim et al. (2020)** [59]	T-47D	MCF-10A	CAP. Plasma device: 0.38 kV, 12.6 mA, 12.9 kHz. Ar gas with 1 L/min flow rate. Exposure times: 30s (10 times), 600 s	qPCR (expression of *ZNRD1* and *ZNRD1-AS1* genes)	(2) Expression of ZNRD1 and ZNRD1-AS1 genes on MCF-10A cells showed significant upregulation of ZNRD1 (*p* < 0.05) and downregulation of ZNRD1-AS1 (*p* < 0.01) after CAP treatment. T-47D cells showed opposite expressions under the same conditions with ZNRD1 downregulated (*p* < 0.01) and ZNRD1-AS1 upregulated (*p* < 0.001). This suggests that CAP regulates ZNRD1, which in turn has a downregulation on ZNRD1-AS1.
**Kumar et al. (2015)** [60]	SK-BR3	HaCaT	APPJ. Plasma device: 60 V, 60 Hz. H_2_O with 400, 600, 800 1000 sccm, and N_2_ gas flow rate is 1 L/min. Exposure times: 0, 1, 3, 5 min Incubation: 24, 48 and 72 h	MTT assay	(1) After 48 h of incubation, SK-BR3 showed reduced viability after 1 min of treatment with N_2_ plasma, with a significant effect after 5 min. N_2_ + H_2_O plasma reduced the viability of cancer cells by 20 to 30% at 5 min of exposure but did not affect HaCaT cells until 48 h.
**Liu et al. (2017)** [52]	MDA-MB-453 MDA-MB-231	MCF-10A	CAP. Plasma device: 10 kHz, 5 mA, Air plasma. Exposure time: 60, 90, 120 s Cells treated with or without NAC and catalase Incubation: 48 h	Trypan blue ROS quantification (DCFH-DA probe, FC) Western blot (IL-6R, pSTAT3, HER2, p-AKT, PTEN expression)	(1) After 120 s of exposure to CAP, the viability of MDA-MB-453 was reduced to less than 80%, while MDA-MB-231 cells fell below 50% (*p* < 0.001) and MCF-10A fell to less than 40% between 60 and 120 s (*p* < 0.001). (2) In MDA-MB-231 cells, NTP treatment led to a decrease in IL-6R and phosphorylated STAT3 (pSTAT3) expression, while in MCF-10A cells, it induced an increase in HER2, phosphorylated Akt (p-AKT), and IL-6R/pSTAT3 levels. (3) MDA-MB-453 cells recovered with NAC, which also reduced the effects of CAP on MCF-10A. Catalase attenuated the effects of CAP on MDA-MB-453 and MCF-10A, although MDA-MB-231 remained vulnerable and strongly inhibited by all treatments. In both cell lines, CAP and NAC reduced pSTAT3 and IL-6R, while increasing PTEN expression.
**Miron et al. (2023)** [61]	MCF-7	MCF-10A	PAS1 results from irradiation of CAP with Ringer’s acetate solution and PAS2 results from irradiation of pyridoxamine solution. Plasma device: 60 Hz, 9 kVp-p. Ar gas with 2.0 SLM flow rate. Dilution of the PAS1 solution: 1, 4, 16, 32, 64 Dilution of the PAS2 solution: 4, 8, 16, 32, 64, 128 Exposure time: 5 min Incubation: 24 h	MTS assay	(1) PAS1 caused cell death in both MCF-7 and MCF-10A lines in dilutions up to 16 times. At 32-fold dilution, MCF7 had 30% viability and MCF-10A 90%. On the other hand, the PAS2 solution was more effective on MCF7 with 14.1% viability at 32-fold dilution and 45.2% at 64-fold dilution.
**Miron et al.** **(2024)** [62]	MCF-7	MCF-10 A	PAS resulted from the irradiation of a chitosan solution diluted in acetic acid. Plasma device: 9 kHz, 9 kVp-p. Ar gas with a flow rate of 10 SLM. Ar combined with O_2_ or N_2_ or N_2_ + O_2_ Exposure time: 5 min Serial dilution rate of the PAS in DMEM: 1×, 2×, 4×, 8×, 16×, 32× Incubation: 24 h	MTS assay	(1) Ar and Ar/O_2_ reduced the viability of malignant cells (10.89 ± 2.7% and 33.98 ± 4.68%, respectively) at a 32-fold dilution, while preserving MCF-10A. With the addition of N_2_ or the Ar/N_2_/O_2_ mixture, cancer cells were effectively eliminated up to a 16-fold dilution. In these conditions, nonmalignant cells remained unaffected, at lower dilutions for N_2_ and a 16-fold dilution for Ar/N_2_/O_2_. Additionally, chitooligosaccharides had minimal effect on MCF-10A cells (86.17 ± 10.37%), while the 25 mg/mL concentration significantly reduced MCF7 viability (17.49 ± 5.97%).
**Mirpour et al. (2014)** [63]	MCF-7	MCF-10A	NAPP jet. Plasma device: 6–10 kV, 6 kHz. He gas with 2 L/min flow rate and He with 5% O_2_. Exposure times: 30, 60, 120, 300 s Other condition: 0.5 mg/mL adriamycin (doxorubicin) drug Incubation: 24 h	MTT assay Apoptotic assay	(1) Exposure of He superior to 30 s reduced the viability of MCF7, without impacting MCF-10A, and compared to treatment with adriamycin, normal cells suffered less damage. Meanwhile, treatment with He/O_2_ caused a higher reduction in the viability of MCF7 (37%) compared to MCF-10A (43%). He NAPP increased apoptosis in cancer cells by up to 3.6 times after 60 s but decreased over time. He/O_2_ NAPP caused apoptosis in the first 30 to 60 s, with necrosis in longer exposures. In MCF-10A, there was no significant damage.
**Misra et al. (2023)** [64]	MCF-7	MCF-10A	CAP. Plasma device: 18.5 V, 11 kHz. Ar flow rate of 18 SLPM and N_2_ flow rate of 0.8 SLPM. Exposure times: 60, 120, 180 s Incubation: 2, 8 and 10 days	Proliferation assay Clonogenic assay	(1) The treatment resulted in a reduction in the survival in both cell lines, and this decrease was more pronounced in MCF7 than in MCF-10A. (2) There was also a reduction in colony formation and a marked differentiation in the induction of DNA damage between the two cell lines, as indicated by the γ-H2AX and 53BP1 markers.
**Mokhtari et al. (2019)** [45]	MCF-7 SKBR3	MCF-10A FMGB-1	PAS. Plasma device: 6 kV. He gas with a 3.5 L/min flow rate. Solution: Medium Exposure times: 2, 4, 6 min Incubation: 48 and 72 h	MTT assay	(1) Longer exposure times reduced cell viability in both cancer lines, especially in SKBR3, with more than 80% death within 6 min at 48 h (*p* < 0.001). In the nonmalignant lines, the same exposure time caused a drop of 5 to 20% (*p* < 0.05 and *p* < 0.01). After 2 min of treatment, viability fell to <50%, with a higher drop at 72 h (*p* < 0.001), and MCF-10A and FMGB-1 had no significant reduction after 2 min.
**Nagaya et al. (2019)** [65]	MDA-MB-231	Human skin fibroblasts	PAS with NAMPT inhibitor, FK866. Ar gas with a flow rate of 2.4 SLM. The distance from the plasma source to the medium surface was 1 mm. Solutions: DMEM without sodium pyruvate. Exposure time: 3 min PAM: 0 and 25 µL/100 µL [FK866]: 0, 1, 10, 100, 1000 nM Incubation: 24 h	MTT assay	(3) MDA-MB-231 cells showed higher cytotoxicity in response to treatment with PAM and FK866, with more pronounced effects at higher concentrations of FK866 and quantity of PAS (*p* < 0.01). In contrast, fibroblasts exhibited a minimal cytotoxic response to FK866 in the presence of PAS (*p* < 0.01).
**Nguyen et al. (2016)** [66]	MDA-MB-231 MDA-MB-453 MDA-MB-468	MCF-10A WI38	PAS. Microplasma jet device with the gas flow rate of 10 L/min. Solution: DMEM Exposure time: 5 min Incubation: 24 h	Live/dead assay	(1) Cell death was different in distinct cancer cell lines: approximately 66% in MDA-MB-453 cells (*p* < 0.001), 59% and 66% in MDA-MB-468 and MDA-MB-231 (*p* < 0.01), respectively. However, cell death was lower in normal cells compared to cancer cells, with 30% in WI38 (*p* < 0.01) and 13% in MCF-10A.
**Ninomiya et al. (2013)** [67]	MCF-7 MDA-MB-231	HMEC	CAP. Plasma device: 0–18 kV, 19 kHz. He plasma jet flow rate at 2 SLM. Exposure time: 600 s Incubation: 24 h	Trypan blue	(1) Cell viability decreased with increasing peak-to-peak voltage for all cell lines. MDA-MB-231 cells were the least affected, followed by MCF-7 cells, while HMECs were the most sensitive. The effective voltages (EV50) were 16.7 ± 0.3 kV for MDA-MB-231, 15.0 ± 0.4 kV for MCF-7, and 11.2 ± 0.7 kV for HMEC.
**Park et al. (2015)** [51]	MCF-7 MDA-MB-231	MCF-10A	CAP. Ar gas with a DBD plasma device. Exposure time: 30 s (10 times) Incubation: 24 h	Annexin V/PI (FC)	(1) Apoptosis increased in both cancer cell lines, being more evident in MDA-MB-231 cells. In contrast, MCF-10A showed no significant changes.
**Pranda et al. (2020)** [68]	MDA-MB-231	MCF-10A	APPJ. Plasma device: 13.5 MHz, 1.2 W, and Ar gas flow of 1.5 SLM. SMD. Plasma device: 42 kHz, 6.5 kV. Exposure time of APPJ: 1 min Exposure time of SMD: 7 min Incubation: 20 h	Live-cell imaging	(2) 1 min with APPJ reduced migration and modified the morphology of cancer cells, without impacting MCF-10A. After 7 min of treatment with APPJ, there were effects on both cell lines, although the treatment with SMD for 7 min only influenced the speed of cell migration.
**Shakya et al. (2022)** [69]	MCF-7	3T3	PAS. Plasma device: 2 kV, 20 kHz. Ar gas flow rate at 2 L/min. Solution: EMEM Exposure times: 1, 2, 3, 4, 5 min Incubation: 48 h	MTT assay	(1) MCF-7 maintained viability in PAS for 1 min and significantly reduced to 16.18% at 5 min (*p* < 0.05). In contrast, the viability of fibroblasts increased by 134.56% in this exposure time (*p* < 0.05).
**Shen et al.** **(2025)** [70]	SUM159PT MCF-7	MCF-10A	PAS supplemented with spermidine. Plasma device: He gas, 10 KHz, 1 L/min. Exposure time: 3.5 min Incubation: 8 h	Viability assay (Cell Counting Kit)	(1) PAS decreased the viability of SUM159PT cells selectively, with no effects in MCF7 and MCF-10A. (3) PAS and spermidine seemed to further enhance SUM159PT proliferation, whereas 1 μM Spd boosted SUM159PT growth.
**Subramanian et al. (2020)** [71]	MDA-MB-231	Murine muscle-derived fibroblast	PAS. Plasma device: 5 kV, 15 kHz, 6.8 ± 0.6 W Solution: Water Volume of PAS activated: 60, 80, 100, 150, 200 mL Exposure times: 6, 12, 18 min Incubation: 24 h	MTT assay	(1) MDA-MB-231 treated with PAS reduced cell viability, with the highest effect at 18 min, which showed a viability of 24% (*p* < 0.001). For fibroblasts, PAS significantly reduced cell viability only at 18 min of exposure (*p* < 0.001).
**Tanaka et al. (2024)** [72]	MDA-MB-231	Fibroblasts	PAS with BPTES, GLS1 inhibitor. The flow rate of Ar gas was at 2.4 SLM. Solution: DMEM Exposure time: 3 min [BTPES]: 0, 2.5, 5 µM [PAM]: 0, 10, 20, 30, 40% Incubation: 24 h	MTT assay	(3) BTPES increased PAM-induced cell death in MDA-MB-231 in a dose-dependent manner (*p* < 0.01). In fibroblasts, pretreatment with BTPES did not affect the cytotoxicity of PAM, but high concentrations of PAM still caused cell death (*p* < 0.05).
**Terefinko et al. (2021)** [73]	MCF-7 MDA-MB-231	MCF-10A	PAS. Plasma device: 2.14 kHz. He gas flow rate of 10.6 L/min. Solutions: DMEM and Opti-MEM medium with or without 3% FBS Exposure times: 150, 180, 210 and 240 s Incubation: 24 h	MTT assay Scratch assay Annexin V/PI (FC)	(1) MCF7 and MDA-MB-231 cells showed a substantial reduction in viability with CAP-activated Opti-MEM, especially with prolonged exposure. After two days, MCF-10A viability increased (*p* < 0.015), whereas MCF-7 and MDA-MB-231 showed higher apoptosis with CAP-activated DMEM and Opti-MEM, with stronger effects on the second day (*p* < 0.0004 and *p* < 0.0015). (2) MCF7 and MDA-MB-231 cells exhibited a significant reduction in migratory capacity after exposure to CAP-activated DMEM and Opti-MEM, especially with prolonged exposures (*p* < 0.002 and *p* < 0.001, respectively). On the other hand, PAS had minimal impact on MCF-10A cells.
**Terefinko et al. (2024)** [53]	MCF-7 MDA-MB-231	MCF-10A	CAP and PAS. Plasma device: 2.14 kHz. He gas was injected into a quartz tube with a 10.6 L/min flow rate. Power: 23.9 ± 0.2 W. Solutions: DMEM and Opti-MEM Exposure times to CAP: 30, 45, 60 s Exposure time of PAM to CAP: 180 s Incubation: 1, 2, 4 and 7 days	MTT assay Scratch assay Annexin V/PI (FC)	(1) CAP reduced cell viability in MCF7 and MCF-10A, depending on the medium and exposure time. In MCF-10A, there was an increase in dead cells in DMEM, but no significant impact in Opti-MEM. In MCF7, CAP did not affect viability in DMEM; however, it was reduced in Opti-MEM after two days. MDA-MB-231 cells were the most affected, with reduced cell survival in both media, mainly due to apoptosis. (2) CAP kept the migration of MCF-10A unchanged, with an increase observed in activated Opti-MEM. In MCF7, there was a slight reduction in migration, while MDA-MB-231 had significantly reduced migration only with CAP in Opti-MEM.
**Wang et al. (2013)** [28]	MDA-MB-231	MSCs	CAP. Plasma device: 60 V/6 A. He flow rate at 4.6 L/min. Exposure times: 30, 60, 90 s Incubation: 1, 3, 5 days	Fluorescence microscopy (live/death viability) MTS assay	(1) MDA-MB231 treated with CAP showed significant inhibition of proliferation, especially after treatments of 60 and 90 s. On the other hand, the MSCs exhibited strong proliferation with CAP treatment compared to the control groups, except after 90 s, which led to a decline in cell density. Fluorescence microscopy with AO/EB staining showed that CAP selectively induced cell death in metastatic BC cells, while sparing healthy mesenchymal stem cells.
**Wang et al. (2020)** [74]	MDA-MB-468 T47D	MCF-10A	CAP combined with NDs. Commercial atmospheric plasma jet (kINPen08). Plasma device: 4.8 kV, Ar plasma jet with a flow rate of 5.0 SLM. Discharge power: 20 W Exposure times: 5, 10, 30, 60 s Incubation: 24 h	Viability assay Confocal microscopy; NDs localization and live/dead cell)	(1) CAP reduced the viability of MDA-MB-468 and T47D cells by 45% and 20%, respectively, after 30 s. (3) CAP combined with NDs reduced the viability of MDA-MB-468 and T47D by 16% to 39%, and MCF-10A remained practically unchanged.
**Wang et al. (2024)** [75]	SUM-159PT MDA-MB-468 MDA-MB-231 HCC70 MCF-7 T-47D	MCF-10A	PAS with EGF. Plasma device: 2–6 kV, 1.7 MHz Ar plasma jet with a flow rate of 5.0 standard L/min. Solutions: DMEM without serum Exposure time: 10 min Incubation: 0.5, 1, 2, 3, 6, 9, 12 and 24 h	Live/dead assay ROS assay ELISA (EGFR resting expression) Western blot (p-EGFR, PLCγ, p-AKT, ERK expression)	(1) Approximately 40% of cell death in MDA-MB-231 and SUM-159PT cells was caused by treatment with 70% 10PAS, while the use of 100% 10PAM led to more than 70% cell death. In MCF-10A, cell death increased at both 12 and 24 h, being more significant at 24 h. (2) CAP treatment increased ROS levels, especially in TNBC cells. Western blot and ELISA analysis showed that EGF stimulated EGFR phosphorylation (Tyr992) and activation of PLCγ and AKT, while 50% 10PAS inhibited AKT phosphorylation, particularly in MDA-MB-231 cells. ERK activation was also elevated in MDA-MB-231 and MCF-10A cells. (3) Treatment with 10PAS and EGF significantly improved the ability to target TNBC cells (MDA-MB-468, -231, and SUM-159PT), although it had less effect on luminal BC cells. ROS levels increased, especially in TNBC cells. EGFR expression was highest in MDA-MB-468, followed by SUM-149PT, SUM-159PT, and MDA-MB-231. MCF7 and MCF-10A showed low EGFR expression. EGFR Tyr992 phosphorylation was enhanced, PLCγ was activated, and phosphorylated AKT was activated by EGF, inhibited by 50% 10PAS, and suppressed in MDA-MB-231. Moreover, ERK activation increased in MDA-MB-231 and MCF-10A.
**Xiang et al. (2018)** [16]	MDA-MB-231 MDA-MB-468 MCF-7	MCF-10A	PAS. Plasma device: 1.0–1.4 kV, 8.8 kHz. He gas with a flow rate of 1 L/min. Solution: Medium Exposure times: 1, 2, 3, 4, 5 min Incubation: 24 h	Viability assay (Cell Counting Kit) Annexin V/PI (FC) Scratch assay	(1) PAS significantly reduced viability and induced cell death in MDA-MB-231 and -468 cells, whereas it slightly increased viability in MCF-10A and MCF7 cells. (2) The migration of MCF-10A and MCF-7 cells was minimally affected over time. Conversely, the two cancer cell lines exhibited a significant reduction in migratory capacity with increasing exposure time (*p* < 0.05).
**Xu et al. (2018)** [76]	SUM-149PT SUM-159PT MDA-MB-436 MDA-MB-231 MCF-7 SKBR3	MCF-10A	PAS. He gas flow rate is 0.5, 1, 1.5 SLM. Plasma device: 1.1, 1.3, 1.5 kV. CAP jet consists of a quartz tube with two conducting electrodes. Solution: RPMI Exposure times: 1, 1.5, 2 min Incubation: 24 h	Viability assay (Cell Counting Kit)	(1) The triple-negative cell lines (SUM149PT, SUM159PT, MDA-MB-231, MDA-MB-436) showed higher viability (70–77%) compared to luminal (MCF7) and HER2-positive (SKBR3) cell lines (34–46%). PAM had a slight effect on MCF-10A under these conditions.

Abbreviations: %—percentage; μM—micromolar; A—amperes; APPJ—atmospheric pressure plasma jet; Ar—argon; ASCs—adipose stem-cells; 53BP1—P53-binding protein 1; BC—breast cancer; BTPES—BPTES (bis-2-(5-phenylacetamido-1, 3, 4-thiadiazol-2-yl) ethyl sulphide); CAP—cold atmospheric plasma; DBD—dielectric barrier device; DCFDA—2’,7’–dichlorofluorescin diacetate; DMEM—Dulbecco’s Modified Eagle Medium; DNA—deoxyribonucleic acid; EGF—epidermal growth factor; EGFR—epidermal growth factor receptor; EMEM—Eagle’s minimum essential medium; ERK—extracellular signal-regulated kinase; FBS—Fetal Bovine Serum; FC—flow cytometry; GLS1—selective glutaminase 1; GSH—reduced glutathione; HaCaT—human nonmalignant keratinocytes; He—helium; HER2—human epidermal growth factor receptor 2; H_2_O—water; HMEC—human mammary epithelial cells; IL-6R—Interleukin-6 receptor; IT50—reduce metabolic activity and viability by 50%; kHz—kilohertz; kV—kilovolts; L—liter; MEM—minimal essential medium; MHz—megahertz; min—minutes; mL—milliliter; mm—millimeter; MSC—mesenchymal stem cells; MTS—3-(4,5-dimethylthiazol-2-yl)-5-(3-carboxymethoxyphenyl)-2-(4-sulfophenyl)-2H-tetrazolium; MTT—3-(4,5-dimethylthiazol-2-yl)-2,5-diphenyl-2H-tetrazolium bromide; N_2_—molecular nitrogen; NAC—N-acetyl cysteine; NAMPT—Nicotinamide phosphoribosyltransferase; NAPP—nonthermal atmospheric pressure plasma; NDs—nanodiamonds; NPs—nanoparticles; O_2_—molecular oxygen; PI—propidium iodide; PLCγ—phospholipase C-gamma; PTEN—phosphatase and tensin homolog; SRB—sulforhodamine B; qPCR—quantitative Polymerase Chain Reaction; ROS—reactive oxygen species; RPMI—Roswell Park Memorial Institute; s—seconds; sccmstandard cubic centimeter per minute; SLM or SLPM—standard liter per minute; SMD—surface micro discharge; SMF—static magnetic fields; STAT3—signal transducer and activator of transcription 3; V—volts; W—watts; WTDF—wild-type dermal fibroblast.

Another study demonstrated that CAP elevated ROS levels in tumor cells (MCF7, AMN3, and AMJ13) while maintaining low ROS levels in nonmalignant cells, indicating that tumor cells become significantly more sensitive to treatment [49].

In studies where cells were exposed to CAP for 5 min, different reductions in cell viability were also seen, where MDA-MB-231 and SK-BR3 showed greater reductions than fibroblasts and HaCaT keratinocytes [58,60].

#### 3.2.2. PAS Treatment

The cell lines MCF-10A, with a normal phenotype, and MCF7 stood out as the most used in the articles included in this review. Other BC cells like HCC1806, AMJ3, MDA-MB-231, SK-BR3, MDA-MB-453, MDA-MB-468, SUM149PT, SUM159PT, MDA-MB-436, 3T3, and WI38, and other nonmalignant cells, like FMGB-1, HFF-1, adipose stem cells, and fibroblasts, were also used. As shown in Table 2, the MTT, SRB, glucose, viability, cell death, scratch, MTS assays, and annexin-V/PI for FC were performed. Different solutions were used for CAP activation, including a range of culture media, water, pyridoxamine, chitosan, and Ringer’s acetate, which had different effects on cells.

Short exposure to plasma-activated cell culture medium revealed that SUM-149PT, SUM-159PT, MDA-MB-436, MDA-MB-231, MCF-7, and SKBR3 cells had better responses with viabilities ranging from 34.4% to 46.4%, and minimal effects on nonmalignant cells [76].

Most studies exposed cell culture media to CAP for longer periods. SKBR3 were highly sensitive to prolonged exposure, with more than 80% cell death after 6 min, whereas MCF-10A and FMGB-1 showed only moderate reductions [45]. Similarly, 5 min of exposure to plasma-activated medium induced significant cell death in MDA-MB-231 (66%), MDA-MB-468 (59%), and MDA-MB-453 (66%), while it had a lesser effect in nonmalignant WI38 (30%) and MCF-10A (13%) cells [66]. Interestingly, another study reported that a 5 min exposure reduced MCF7 viability to 16.18%; however, it increased 3T3 fibroblasts by 134.56% [69]. On the other hand, 3.5 min of plasma-activated medium exposure decreased the viability of SUM159PT cells, while MCF7 and MCF-10A cells remained unaffected [70]. Thus, the results obtained by Terefinko et al. support the previous outcomes described. They concluded that the effectiveness of the treatment varied according to the medium used; for instance, activated DMEM had more effect on MCF-10A and MDA-MB-231 cells, while activated Opti-MEM affected MCF7 [53].

It is known that CAP and plasma-activated cell culture medium can affect cell migration. Migration was significantly reduced in MCF7 and MDA-MB-231 cell lines, while minimal effects were seen on MCF-10A [53,73]. This was corroborated by Xiang et al.’s findings that demonstrated a significant reduction in cancer cell migration, MDA-MB-231, and MDA-MB-468, with a slight effect on MCF-10A and MCF7 [16]. Plasma-activated medium also raised glucose levels in MCF7 and AMJ13 cells, while it had less effect on adipose stem cells [56].

Similar to CAP, plasma-activated medium led to greater ROS production in TNBC cells than in non-TNBC and MCF10A cells. Moreover, ROS scavengers reinstated TNBC cell viability, suggesting CAP selectivity is dependent on ROS, including superoxide, hydrogen peroxide, hydroxyl radical, and nitric oxide [55].

Regarding other activated solutions, water lowered MDA-MB-231 viability to 24% after 18 min, also with less effect on fibroblasts [71]. Ringer’s acetate solution showed a high cytotoxic profile with both malignant and nonmalignant cell death [61]. Miron et al. established that different dilutions of plasma-activated chitosan and gases resulted in the death of BC cells and the survival of nonmalignant cells [62].

#### 3.2.3. Combination of CAP or PAS Treatment with Other Therapies

MDA-MB-231, MCF7, and MCF-10A cells were the most frequently used in articles reporting combinatory studies. Techniques such as MTT, viability, ROS quantification, seahorse, wound healing assay, live/dead, apoptotic assays, ELISA, western blot, confocal microscopy, and annexin V/PI (FC) were used to evaluate the results. CAP or PAM were applied in combination with static magnetic fields (SMF), nanoparticles, NAMPT (nicotinamide phosphoribosyltransferase, FK866) or GLS1 (selective glutaminase 1, BPTES) inhibitors, epidermal growth factor (EGF), spermidine, and drugs like atorvastatin.

Combination strategies of CAP and other moieties show the potential to improve cancer cell response, while, in several cases, being protective of nonmalignant cells. For instance, CAP (30 s) reduced cell viability by 15% in WTDF and 60% in MDA-MB-231 cells, with SMF further increasing MDA-MB-231 death by up to 25%, and no effects in WTDF [54]. Also, CAP (30–45 s) and iron nanoparticles per se significantly reduced MCF7 viability, with combined treatments being more effective, *p* < 0.001, while fibroblasts remained unaffected [57]. Similar results were demonstrated regarding the viability of MDA-MB-468 and T47D vs. MCF-10A when CAP (60 s) was combined with nanodiamonds [74]. The combination of CAP and atorvastatin also improved anticancer efficacy, promoting positive effects on tumor growth, apoptosis, and cell migration [55].

Nonmalignant MCF-10A cells were more susceptible to ROS than TNBC after CAP exposure. However, NAC and catalase protected MCF-10A cells, with NAC being effective at lower ROS levels and catalase at higher ones [52]. This suggests that combining CAP with antioxidants may protect nonmalignant cells without reducing its anticancer effect [52].

Combinations of PAS and soluble factors were also investigated. Association of PAS with BTPES, a selective glutaminase inhibitor1, and FK866, a nicotinamide phosphoribosyltransferase inhibitor, led to an increase in cytotoxicity in MDA-MB-231 cells with a slight effect on fibroblasts [65,72]. Conversely, PAS and spermidine further increased proliferation and growth in SUM159PT and MCF7, slightly reducing viability in MCF-10A cells [70]. Also, the combination of PAS and EGF increased ROS levels and caused up to 70% cell death in TNBC cells, with the greatest effect on MDA-MB-468, due to the intensification of epidermal growth factor receptor (EGFR) phosphorylation and phospholipase C-gamma (PLCγ) activation [75]. Treatment with PAS inhibited AKT phosphorylation, while extracellular signal-regulated kinase (ERK) activation increased in MDA-MB-231 and MCF-10A [75].

### 3.3. Risk of Bias Evaluation

**Figure 3:**
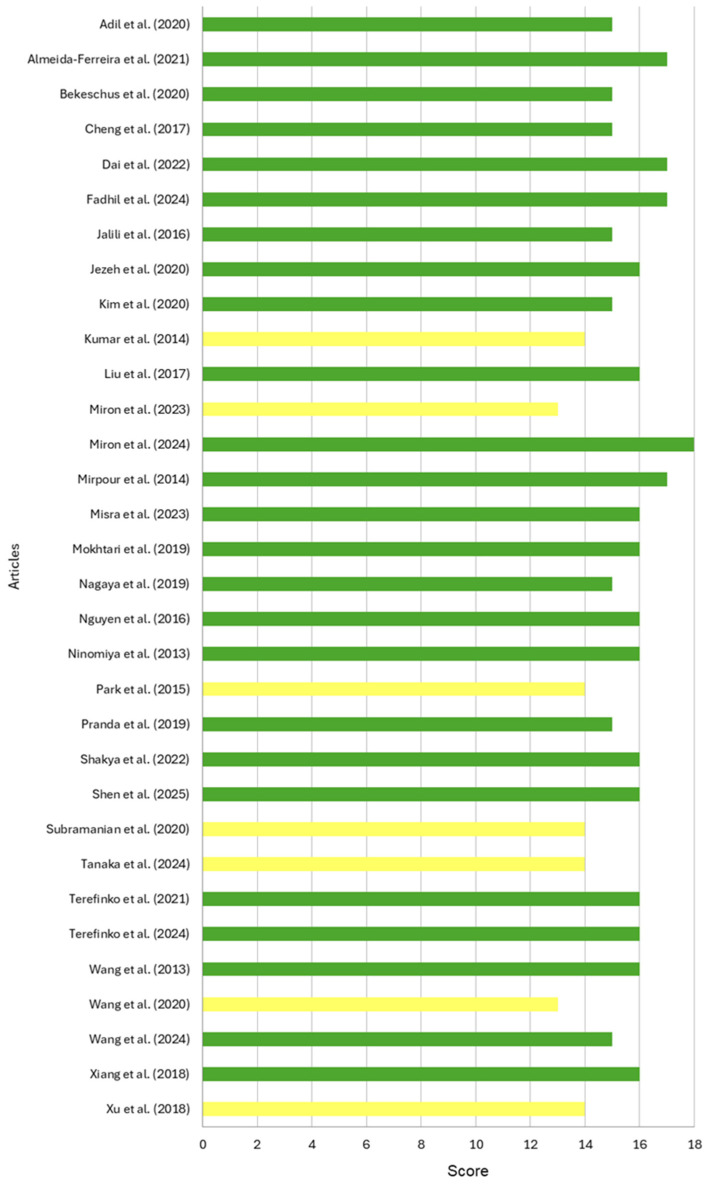
Quality assessment scores of the in vitro studies included in the systematic review were evaluated using the Toxicological Data Reliability Assessment Tool (ToxRTool). Studies with scores from 11 to 14 [51,60,61,71,72,74,76] and 15 to 18 [16,17,28,45,49,50,52,53,54,55,56,57,58,59,62,63,64,65,66,67,68,69,70,73,75] were represented in yellow and green, respectively.

The quality of the studies was assessed using the ToxRTool, and the articles were classified according to the criteria of this protocol. Figure 3 shows the analysis of the risk of bias in each study, emphasizing that the predominant scores ranged from 15 to 18, indicating that most of the studies were considered reliable without restrictions. Moreover, just 7 articles were classified as reliable with some restrictions (scores between 11 and 14 in this systematic review; no article was rated lower than a score of 11). The risk assessment results are detailed in Supplementary Materials S2—Table S1.

**Table 3:** Summary of cell viability reduction.

			Nonmalignant Cells		Breast Cancer Cells	
	Treatment	Assay	Cells	Reduction in Cell Viability (%)	Cell Line	Reduction in Cell Viability (%)
Almeida-Ferreira et al. (2021) [17]	CAP	MTT	HFF-1	50	HCC1806	50
					MCF-7	50
Cheng et al. (2017) [54]	CAP	MTT	WTDF	15	MDA-MB-231	60
Jezeh et al. (2020) [58]	CAP	MTT	HF	30	MDA-MB-231	60
	PAS			17		50
Liu et al. (2017) [52]	CAP	Trypan blue	MCF10-A	>60	MDA-MB-231	>50
Miron et al. (2023) [61]	PAS	MTS	MCF10-A	10	MDA-MB-453	>20
Miron et al. (2024) [62]	PAS	MTS	MCF10-A	7.4	MCF-7	96.6
Shakya et al. (2022) [69]	PAS	MTT	3T3	−34.6	MCF-7	83.8
Xiang et al. (2018) [16]	PAS	Cell viability (Cell Counting Kit)	MCF10-A	−16	MDA-MB-231	59
					MDA-MB-468	54
					MCF-7	−44

CAP—Cold atmospheric plasma; PAS—Plasma-activated solution.

## 4. Discussion

In the last few years, cancer has become a major global public health concern, largely due to the progressive aging of the population, which helps explain the considerable increase in both incidence and mortality rates worldwide [4,77]. Conventional anti-tumor therapies applied in clinical practice have several shortcomings, including low selectivity, the development of drug resistance, rapid metabolization, high tumor recurrence rates, and significant adverse effects [78]. Given the effects of CAP across various medical fields, particularly in tissue regeneration, dentistry, and chronic or acute wound healing, it has recently been proposed as a potential anti-tumor therapy, especially for BC [40]. Moreover, several in vitro and in vivo studies have demonstrated CAP’s selective cytotoxicity with promising results for several cancer types [18,26,29,79,80]. Tanaka et al. reported that PAS increased apoptosis in glioblastoma cells without affecting nonmalignant astrocytes [81]. Similarly, CAP reduced proliferation in head and neck cancer cells compared to keratinocytes and significantly decreased prostate cancer cells’ survival while preserving nonmalignant cells [82,83]. Considering all this evidence and that CAP’s efficacy and safety are still under preclinical evaluation [84], this systematic review aimed to determine whether CAP and PAS exhibit in vitro selectivity for BC cells.

The gathered evidence indicates that the experimental outcomes depend on the settings of each protocol, including the exposure time, the treatment approach (CAP or PAS), and the settings of the irradiation device. A wide variety of evaluation techniques have been used, including MTT, MTS, SRB, annexin-V/propidium iodide, trypan blue, viability, scratch, ROS quantification, clonogenic, resazurin, and antioxidant defense assays, to investigate the biological specificity of CAP’s effects on cultured cells. Among these studies, exposure times of 60 and 120 s were the most common [16,17,28,45,50,52,53,54,55,58,60,63,64,68,69,74,76]. Both CAP and PAS generally demonstrated a stronger tendency to reduce the viability of BC cells than of nonmalignant cells [16,17,50,52,54,58,61,63,69]. These treatments also exhibited a tendency to reduce the migration of BC cells, with minimal impact on nonmalignant cells [16,53,68,73], which can be attributed to the high basal ROS levels typically present in cancer cells [49].

Over the years, several mechanisms have been proposed to explain the selective susceptibility of cancer cells to CAP. Biochemically, malignant cells often display elevated basal ROS levels due to metabolic reprogramming and dysregulated redox homeostasis. CAP exposure further elevates ROS and RONS beyond the buffering capacity of cellular antioxidant defenses, precipitating apoptosis. Effectively, susceptibility to oxidative stress is compounded by upregulated aquaporins and NADPH oxidases (NOX), which enhance RONS influx and endogenous ROS production, and by reduced membrane cholesterol, which increases bilayer fluidity and poration proneness [43,49,55,85]. From a physicochemical perspective, CAP’s ionized gases and charged particles induce transient electric fields that initiate nanoscale structural changes in the cell membrane, creating transient poration in the lipid bilayer, increasing membrane permeability. Thus, it has been suggested that CAP may facilitate the entry of RONS into the cell, where they induce oxidative damage to lipids, proteins, and DNA, impair mitochondrial function, and trigger cell-cycle arrest and ultimately drive tumor cell death [17]. This mechanism may help explain the selective inhibition of TNBC cell proliferation and migration, while promoting MSC proliferation and not affecting the migration of nonmalignant cells [28,53].

The effects of CAP or PAS have also been studied in combination with other treatments, such as nanoparticles, atorvastatin, and BTPES. These studies demonstrated that CAP increased the effectiveness of these therapeutic approaches [55,57,72].

The hypothesis of this systematic review was that CAP and PAS selectively induced cytotoxicity in BC cells. Several studies on other cancer types also support this hypothesis, as CAP or PAS selectivity has been reported previously. For instance, a study on ovarian cancer cell lines revealed that exposure to CAP decreased the proliferation rates of these cells, in comparison with fibroblast cells [86]. Similarly, another study showed that exposing cells to CAP induced increased apoptosis in melanoma cells compared to keratinocytes [87]. However, due to the great heterogeneity of the results among the included articles, encompassing the diversity of cell types, treatment sources, administration methods, and exposure times, it is not possible to robustly confirm our hypothesis. The key parameters, such as applied voltage, gas flow rate, and solution type, may influence how tumor and nonmalignant cells respond to CAP. We hypothesize that the applied voltage can affect the amount of RONS produced, as higher voltages generate enough RONS to overwhelm tumor cells, which already experience elevated oxidative stress, leading to selective cytotoxicity, while lower voltages may not produce sufficient RONS to induce clear differences. Similarly, the gas flow rate can modify how RONS are delivered to the cells, with higher flow potentially increasing RONS exposure but also altering radical stability and uptake, which may affect cell-specific responses. Finally, the type of solution used, such as culture medium versus buffer, can influence RONS stability and reactivity, since proteins, ions, and antioxidants in the medium may scavenge or transform RONS, changing the oxidative load experienced by the cells. Together, these factors likely contribute to variability in reported selectivity and highlight the importance of careful control and detailed reporting of experimental conditions in future CAP studies. According to the data presented in Table 2, 71.88% of the studies reported that CAP exhibited selectivity toward BC cells, whereas 28.12% demonstrated only partial selectivity. In these latter cases, CAP was less effective or had no significant effect on certain tumor cell lines, showing minimal differences compared to the control condition. Another limitation in the interpretation of selectivity could be the biological variability of the nonmalignant cell models used across the different studies (such as MCF-10A, fibroblasts, keratinocytes, and mesenchymal stem cells). These cells differ substantially in their biological characteristics and tolerance to oxidative stress, which may influence their response to CAP exposure and thus limit the generalizability of the observed selective effects.

To reduce bias and improve the reliability of comparisons, we selected only studies that simultaneously evaluated both BC and nonmalignant cells under similar experimental conditions and methodologies, allowing more reliable within-study comparisons. Additionally, studies using the same cell lines and identical exposure times could be compared more accurately through statistical analysis to assess the effects of CAP. Although most results included in this systematic review could be compared, a few studies provided limited descriptions of CAP characteristics and their effects on the cell lines used, as well as due to the significant variability in CAP sources, which made it impossible to carry out the planned meta-analysis. These challenges are further supported by the quality assessment of the articles described in Figure 3. Most of the articles included in this systematic review reported a score above 14 points, which corroborates the reliability of the included results. Studies with lower quality scores were included to provide a comprehensive overview of the literature; however, their limitations were carefully considered. Nevertheless, to mitigate the variability of the results from CAP or PAS studies, particularly given their emerging status as anti-tumoral therapies, strategies such as standardizing experimental protocols, using validated methodologies, employing consistent and well-characterized models, conducting multidisciplinary collaborative trials, ensuring comprehensive reporting, and establishing consensus guidelines are crucial for enhancing comparative outcomes and scientific rigor. Nevertheless, CAP and PAS treatment have emerged as a potential selective therapeutic option for breast tumor cells.

As a future perspective, the biological effects should be deepened, namely the signaling pathways inherent to the differentiated responses of this treatment in BC cells and nonmalignant cells.

## 5. Conclusions

The CAP and PAS treatments have demonstrated promising potential in selectively targeting BC cell lines while sparing nonmalignant cells in in vitro studies. Current evidence suggests that CAP may exhibit a favorable non-cytotoxic profile for nonmalignant cells and healthy tissues, with limited reports of significant toxicity under comparable exposure times and conditions. These findings highlighted its potential as a novel anti-tumoral therapeutic strategy with important advantages for BC management. Moreover, combining CAP with other treatment modalities has demonstrated the potential for enhancing therapeutic efficacy without compromising CAP’s selectivity. Nonetheless, further research is essential to elucidate the mechanisms underlying CAP’s selectivity. In particular, the role of ROS is considered to have distinct characteristics of cancer cells, such as elevated baseline ROS levels, increased expression of aquaporins, and altered antioxidant defense systems, compared to nonmalignant cells. Moreover, exploring other possible mechanisms through comprehensive in vitro and in vivo studies performed under diverse conditions with standardized protocols. Additionally, evaluating the long-term cytotoxicity and potential adverse effects of repeated CAP exposure in nonmalignant cells, including the risks of DNA damage and mutagenesis, is crucial through in vivo studies and robust clinical trials.

## Figures and Tables

**Table 1:** Research question structure using the population, intervention, comparison, and outcome (PICO) framework.

Parameter	Description
Population (P)	Breast cancer and nonmalignant cells
Intervention (I)	Cold atmospheric plasma
Comparison (C)	None
Outcome (O)	Cell viability

## Data Availability

The data presented in this study are available in the manuscript.

## Supplementary Materials

The supporting information can be downloaded at: https://www.mdpi.com/article/10.3390/ijms27041710/s1.

## Abbreviations

The following abbreviations are used in this manuscript:

ATP	Adenosine triphosphate
BC	Breast cancer
BTPES	bis-2-(5-phenylacetamido-1, 3, 4-thiadiazol-2-yl) ethyl sulphide
CAP	Cold atmospheric plasma
DBD	Dielectric barrier discharge
DNA	Deoxyribonucleic acid
EGF	Epidermal growth factor
EGFR	Epidermal growth factor receptor
ER	Estrogen receptors
ERK	Extracellular signal-regulated kinase
FC	Flow cytometry
GLS1	Selective glutaminase 1
GSH	Reduced glutathione
HER2	Human epidermal growth factor receptor type 2
MTS	[3-(4,5 dimethylthiazol-2-yl)-5-(3-carboxymethoxyphenyl)-2-(4-sulfophenyl)-2H-tetrazolium]
MTT	3-(4,5-dimethylthiazol-2-yl)-2,5-diphenylte trazolium bromide; NAMPT, nicotinamide phos-phoribosyltransferase
NRF2	Nuclear factor erythroid 2-related factor 2
OSF	Open Science Framework Registrations
PAA	Plasma-activated Ringer’s acetate solution
PAC	Plasma-activated chitosan
PAM	Plasma-activated media
PAS	Plasma-activated solution
PAW	Plasma-activated water
PI	Propidium iodide
PICO	Population, intervention, comparison, and outcome
PLCγ	Phospholipase C-gamma
PR	Progesterone receptors
PRISMA	Preferred Reporting Items for Systematic Reviews and Meta-Analyses
qPCR	Quantitative polymerase chain reaction
RONS	Reactive oxygen and nitrogen species
ROS	Reactive oxygen species
s	seconds
SMF	Static magnetic fields
SRB	Sulforhodamine B
TNBC	Triple-negative breast cancer
TNM	Tumor-Node-Metastasis
ToxRTool	Toxicological Data Reliability Assessment Tool
UV	Ultraviolet
WHO	World Health Organization
