# Supplementary material for: Is Cold Atmospheric Plasma Selective for Breast Tumor Cells? A Systematic Review
Source: Int J Mol Sci. 2026 Feb 10;27(4):1710. doi: 10.3390/ijms27041710 (PMC12940492; doi:10.3390/ijms27041710)
Supplement: Supplementary file 1 [file ijms-27-01710-s001.zip › ijms-4109859-supplementary.pdf]

Supplementary material

S1. Literature Search

1.1. Medline (through PubMed)

("Breast Neoplasms"[Mesh] OR “Breast Neoplas*” OR “Neoplasm, Breast” OR “Neoplasms, Breast” OR “breast neoplastic cell line” OR “neoplastic mammary cell line” OR “neoplastic breast” OR “malignant neoplasm of breast” OR “breast malignant neoplasm*” OR “breast gland neoplasm” OR “neoplasia of the breast” OR “neoplasm of the breast” OR “Breast Tumor*” OR “Tumor, Breast” OR “Tumors, Breast” OR “breast tumor-derived cell line” OR “tumoral breast cell line” OR “tumor of the breast” OR “tumor of the female breast” OR “tumor of the male breast” OR “tumorigenesis of the breast” OR “tumorigenic breast cell line” OR “breast gland tumor” OR “Breast Cancer*” OR “Cancer, Breast” OR “cancer of breast” OR “ca breast” OR “breast gland cancer” OR “cancer of the breast” OR “Malignant Tumor of Breast” OR “Breast Malignant Tumor*” OR “Cancer of the Breast” OR “Cancer of Breast” OR “Malignant Neoplasm of Breast” OR “Breast Malignant Neoplasm*” OR “Mammary Cancer*” OR “Cancer, Mammary” OR “Cancers, Mammary” OR “mamma cancer” OR “mammary gland cancer” OR “cancer in the mammary gland” OR “cancer of the mammary gland” OR “Mammary Carcinoma, Human” OR “Carcinoma, Human Mammary” OR “Carcinomas, Human Mammary” OR “Human Mammary Carcinoma*” OR “Mammary Carcinomas, Human” OR “carcinoma in the mammary gland” OR “carcinoma of the mamma” OR “carcinoma of the mammary gland” OR “carcinoma, mammary” OR “carcinomatous mammary” OR “mamma carcinoma*” OR “mammary carcinoma*” OR “mammary gland carcinoma” OR “mammary carcinoma-derived cell line” OR “Mammary Neoplasm*” OR “Neoplasm, Human Mammary” OR “Neoplasms, Human Mammary” OR “Mammary Neoplasm, Human” OR “mammary gland neoplas*” OR “mammary neoplasia” OR “neoplasm of the mammary gland” OR “neoplastic mammary” OR “mammary tumor*” OR “mammary tumor-derived cell line” OR “mamma tumor” OR “mammary gland tumor*” OR “tumor of the mammary gland” OR “tumorigenesis of the mammary gland” OR “mammary gland malignancy” OR “mammary malignanc*” OR “Breast Carcinoma*” OR “Carcinoma, Breast” OR “Carcinomas, Breast” OR “carcinoma of the breast” OR “carcinomata of the breast” OR “breast carcinoma-derived cell line” OR “carcinomatous breast” OR “breast malignanc*” OR “malignancies of the breast” OR “malignancy of the breast” OR “breast mass” OR “mass in the breast” OR “masses in the breast”) AND ("Plasma Gases"[Mesh] OR “Plasma gases” OR “plasma gas” OR “Gases, Plasma” OR “Cold Plasma” OR “Plasma, Cold” OR “Non-Thermal Atmospheric Pressure Plasma” OR “Non Thermal Atmospheric Pressure Plasma” OR “Thermal Plasma” OR “Plasma, Thermal” OR “cold atmospheric plasma”)

Filters: Language: Portuguese, Spanish, French, English.

1.2. Web of Science (all databases)

(“Breast Neoplas*” OR “Neoplasm, Breast” OR “Neoplasms, Breast” OR “breast neoplastic cell line” OR “neoplastic mammary cell line” OR “neoplastic breast” OR “malignant neoplasm of breast” OR “breast malignant neoplasm*” OR “breast gland neoplasm” OR “neoplasia of the breast” OR “neoplasm of the breast” OR “Breast Tumor*” OR “Tumor, Breast” OR “Tumors, Breast” OR “breast tumor-derived cell line” OR “tumoral breast cell line” OR “tumor of the breast” OR “tumor of the female breast” OR “tumor of the male breast” OR “tumorigenesis of the breast” OR “tumorigenic breast cell line” OR “breast gland tumor” OR “Breast Cancer*” OR “Cancer, Breast” OR “cancer of breast” OR “ca breast” OR “breast gland cancer” OR “cancer of the breast” OR “Malignant Tumor of Breast” OR “Breast Malignant Tumor*” OR “Cancer of the Breast” OR “Cancer of Breast” OR “Malignant Neoplasm of Breast” OR “Breast Malignant Neoplasm*” OR “Mammary Cancer*” OR “Cancer, Mammary” OR “Cancers, Mammary” OR “mamma cancer” OR “mammary gland cancer” OR “cancer in the mammary gland” OR “cancer of the mammary gland” OR “Mammary Carcinoma, Human” OR “Carcinoma, Human Mammary” OR “Carcinomas, Human Mammary” OR “Human Mammary Carcinoma*” OR “Mammary Carcinomas, Human” OR “carcinoma in the mammary gland” OR “carcinoma of the mamma” OR “carcinoma of the mammary gland” OR “carcinoma, mammary” OR “carcinomatous mammary” OR “mamma carcinoma*” OR “mammary carcinoma*” OR “mammary gland carcinoma” OR “mammary carcinoma-derived cell line” OR “Mammary Neoplasm*” OR “Neoplasm, Human Mammary” OR “Neoplasms, Human Mammary” OR “Mammary Neoplasm, Human” OR “mammary gland neoplas*” OR “mammary neoplasia” OR “neoplasm of the mammary gland” OR “neoplastic mammary” OR “mammary tumor*” OR “mammary tumor-derived cell line” OR “mamma tumor” OR “mammary gland tumor*” OR “tumor of the mammary gland” OR “tumorigenesis of the mammary gland” OR “mammary gland malignancy” OR “mammary malignanc*” OR “Breast Carcinoma*” OR “Carcinoma, Breast” OR “Carcinomas, Breast” OR “carcinoma of the breast” OR “carcinomata of the breast” OR “breast carcinoma-derived cell line” OR “carcinomatous breast” OR “breast malignanc*” OR “malignancies of the breast” OR “malignancy of the breast” OR “breast mass” OR “mass in the breast” OR “masses in the breast”) AND (“Plasma gases” OR “plasma gas” OR “Gases, Plasma” OR “Cold Plasma” OR “Plasma, Cold” OR “Non-Thermal Atmospheric Pressure Plasma” OR “Non Thermal Atmospheric Pressure Plasma” OR “Thermal Plasma” OR “Plasma, Thermal” OR “cold atmospheric plasma”)

Filters: NOT document types: Retracted Publication or Abstract or Awarded Grant or Meeting or Editorial Material or Review Article or Clinical Trial; NOT Languages: Korean.

1.3. Embase

('breast cancer'/exp OR 'breast cancer*' OR 'cancer of breast' OR 'ca breast' OR 'breast gland cancer' OR 'cancer of the breast' OR 'cancer, breast' OR 'breast neoplas*' OR 'neoplasm, breast' OR 'neoplasms, breast' OR 'breast neoplastic cell line' OR 'neoplastic mammary cell line' OR 'neoplastic breast' OR 'malignant neoplasm of breast' OR 'breast malignant neoplasm*' OR 'breast gland neoplasm' OR 'neoplasia of the breast' OR 'neoplasm of the breast' OR 'breast tumor'/exp OR 'breast tumor*' OR 'tumor breast' OR 'tumors, breast' OR 'breast tumor cell line'/exp OR 'breast tumor-derived cell line' OR 'tumoral breast cell line' OR 'tumor of the breast' OR 'tumor of the female breast' OR 'tumor of the male breast' OR 'tumorigenesis of the breast' OR 'tumorigenic breast cell line' OR 'malignant tumor of breast' OR 'breast malignant tumor*' OR 'breast gland tumor' OR 'breast carcinoma'/exp OR 'breast carcinoma*' OR 'carcinoma of the breast' OR 'carcinomata of the breast' OR 'carcinoma, breast' OR 'carcinomas, breast' OR 'breast carcinoma cell line'/exp OR 'breast carcinoma-derived cell line' OR 'carcinomatous breast' OR 'breast malignanc*' OR 'malignancies of the breast' OR 'malignancy of the breast' OR 'malignant neoplasm of the breast' OR 'breast mass' OR 'mass in the breast' OR 'masses in the breast' OR 'mammary cancer*' OR 'cancer, mammary' OR 'cancers, mammary' OR 'mamma cancer' OR 'mammary gland cancer' OR 'cancer in the mammary gland' OR 'cancer of the mammary gland' OR 'mammary carcinoma, human' OR 'mammary carcinomas, human' OR 'carcinoma, human mammary' OR 'carcinomas, human mammary' OR 'carcinoma in the mammary gland' OR 'carcinoma of the mamma' OR 'carcinoma of the mammary gland' OR 'carcinoma, mammary' OR 'human mammary carcinoma*' OR 'carcinomatous mammary' OR 'mamma carcinoma*' OR 'mammary carcinoma*' OR 'mammary gland carcinoma' OR 'mammary carcinoma-derived cell line' OR 'mammary neoplasm*' OR 'neoplasm, human mammary' OR 'neoplasms, human mammary' OR 'mammary gland neoplas*' OR 'mammary neoplasia' OR 'neoplasm of the mammary gland' OR 'neoplastic mammary' OR 'mammary tumor*' OR 'mammary tumor-derived cell line' OR 'mamma tumor' OR 'mammary gland tumor*' OR 'tumor of the mammary gland' OR 'tumorigenesis of the mammary gland' OR 'mammary gland malignancy' OR 'mammary malignanc*') AND ('plasma gas'/exp OR 'plasma gas' OR 'plasma gases' OR 'gases, plasma' OR 'cold plasma'/exp OR 'cold plasma' OR 'plasma, cold' OR 'non-thermal atmospheric pressure plasma' OR 'non thermal atmospheric pressure plasma'/exp OR 'thermal plasma' OR 'plasma, thermal' OR 'cold atmospheric plasma'/exp OR 'cold atmospheric plasma') AND ([english]/lim OR [french]/lim OR [portuguese]/lim OR [spanish]/lim) AND ([article]/lim OR [article in press]/lim OR [data papers]/lim OR [letter]/lim)

1.4. Cochrane Library

#1 MeSH descriptor: [Plasma Gases] explode all trees 51

#2 "Plasma Gases" 52

#3 "plasma gas" 31

#4 "Gases, Plasma" 11

#5 "Cold Plasma"32

#6 "Plasma, Cold" 2

#7 "Non-Thermal Atmospheric Pressure Plasma" 6

#8 "Non Thermal Atmospheric Pressure Plasma" 6

#9 "Thermal Plasma" 6

#10 "Plasma, Thermal" 0

#11 "cold atmospheric plasma" 51

#12 #1 OR #2 OR #3 OR #4 OR #5 OR #6 OR #7 OR #8 OR #9 OR #10 OR #11 138

#13 MeSH descriptor: [Breast Neoplasms] explode all trees 20230

#14 (Breast NEXT neoplas*) 20826

#15 (neoplasm* NEXT breast) 679

#16 "breast neoplastic cell line" 0

#17 "neoplastic mammary cell line" 0

#18 "neoplastic breast" 4

#19 "malignant neoplasm of breast" 760

#20 (breast malignant NEXT neoplasm*) 1449

#21 "breast gland neoplasm" 0

#22 "neoplasia of the breast" 0

#23 "neoplasm of the breast" 14

#24 (breast NEXT tumor*) 1819

#25 (tumor* NEXT breast) 805

#26 "breast tumor-derived cell line" 0

#27 "tumoral breast cell line" 0

#28 "tumor of the breast" 6

#29 "tumor of the female breast" 0

#30 "tumor of the male breast" 0

#31 "tumorigenesis of the breast" 0

#32 "tumorigenic breast cell line"0

#33 "breast gland tumor" 0

#34 (breast NEXT cancer*) 43800

#35 "cancer, breast" 1749

#36 "cancer of breast" 12

#37 "ca breast" 24

#38 "breast gland cancer" 0

#39 "cancer of the breast" 79

#40 "Malignant Tumor of Breast" 1

#41 (Breast Malignant NEXT Tumor*) 181

#42 "cancer of the breast" 79

#43 "cancer of breast" 12

#44 "malignant neoplasm of breast" 760

#45 (breast malignant NEXT neoplasm*) 1449

#46 (mammary NEXT cancer*) 63

#47 (cancer* NEXT mammary) 3

#48 "mamma cancer" 1

#49 "mammary gland cancer" 0

#50 "cancer in the mammary gland" 0

#51 "cancer of the mammary gland" 2

#52 (mammary carcinoma* NEXT human) 1

#53 (carcinoma* NEXT human mammary) 1

#54 (human mammary NEXT carcinoma*) 20

#55 "carcinoma in the mammary gland" 0

#56 "carcinoma of the mamma" 0

#57 "carcinoma of the mammary gland" 0

#58 "carcinoma, mammary" 2

#59 "carcinomatous mammary" 0

#60 (mamma NEXT carcinoma*) 33

#61 (mammary NEXT carcinoma*) 102

#62 "mammary gland carcinoma"5

#63 "mammary carcinoma-derived cell line" 0

#64 (Mammary NEXT Neoplasm*) 39

#65 (mammary neoplasm* NEXT human) 2

#66 (human mammary NEXT neoplasm*) 22

#67 (neoplasm* NEXT human mammary) 2

#68 (mammary gland NEXT neoplas*) 2

#69 "mammary neoplasia" 1

#70 "neoplasm of the mammary gland" 1

#71 "neoplastic mammary" 0

#72 (mammary NEXT tumor*) 43

#73 "mammary tumor-derived cell line" 0

#74 "mamma tumor" 0

#75 (mammary gland NEXT tumor*) 5

#76 "tumor of the mammary gland" 0

#77 "tumorigenesis of the mammary gland" 0

#78 "mammary gland malignancy" 0

#79 (mammary NEXT malignanc*) 0

#80 (breast NEXT carcinoma*) 2309

#81 (carcinoma* NEXT breast) 190

#82 "carcinoma of the breast" 286

#83 "carcinomata of the breast" 12

#84 "breast carcinoma-derived cell line" 0

#85 "carcinomatous breast" 0

#86 (breast NEXT malignanc*) 102

#87 "malignancies of the breast" 6

#88 "malignancy of the breast" 4

#89 "breast mass" 66

#90 "mass in the breast" 1

#91 "masses in the breast" 2

#92 #13 OR #14 OR #15 OR #16 OR #17 OR #18 OR #19 OR #20 OR #21 OR #22 OR #23 OR #24 OR #25 OR #26 OR #27 OR #28 OR #29 OR #30 OR #31 OR #32 OR #33 OR #34 OR #35 OR #36 OR #37 OR #38 OR #39 OR #40 OR #41 OR #42 OR #43 OR #44 OR #45 OR #46 OR #47 OR #48 OR #49 OR #50 OR #51 OR #52 OR #53 OR #54 OR #55 OR #56 OR #57 OR #58 OR #59 OR #60 OR #61 OR #62 OR #63 OR #64 OR #65 OR #66 OR #67 OR #68 OR #69 OR #70 OR #71 OR #72 OR #73 OR #74 OR #75 OR #76 OR #77 OR #78 OR #79 OR #80 OR #81 OR #82 OR #83 OR #84 OR #85 OR #86 OR #87 OR #88 OR #89 OR #90 OR #91 47150

#93 #12 AND #92 1

S2. Quality analysis

Table S1 - Quantitative details of questions from the ToxRTool tool.

Study	**1. Was the test substance identified?**	**2. Is the purity of the substance given?**	**3. Is information on the source/origin of the substance given?**	**4. Is all information on the nature and/or physico-chemical properties of the test item given, which you deem indispensable for judging the data (see explanation for examples)?**	**5. Is the test system described?**	**6. Is information given on the source/origin of the test system?**	**7. Are necessary information on test system properties, and on conditions of cultivation and maintenance given?**	**8. Is the method of administration given (see explanations for details)?**	**9. Are doses administered or concentrations in application media given?**	**10. Are frequency and duration of exposure as well as time-points of observations explained?**	**11. Were negative controls included (give also point, if not necessary, see explanations)?**	**12. Were positive controls included (give also point, if not necessary, see explanations)?**	**13. Is the number of replicates (or complete repetitions of experiment) given?**	**14. Are the study endpoint(s) and their method(s) of determination clearly described?**	**15. Is the description of the study results for all endpoints investigated transparent and complete?**	**16. Are the statistical methods for data analysis given and applied in a transparent manner (give also point, if not necessary/applicable, see explanations)?**	**17. Is the study design chosen appropriate for obtaining the substance-specific data aimed at (see explanations for details)?**	**18. Are the quantitative study results reliable (see explanations for arguments)?**	TOTAL
	Test substance identification				Test system characterization			Study design description						Study results documentation			Plausibility of study design and data		
Adil et al. (2020)	1	1	1	1	1	1	1	1	1	1	1	0	1	1	0	0	1	1	15
Almeida-Ferreira et al. (2021)	1	1	1	1	1	1	1	1	1	1	1	0	1	1	1	1	1	1	17
Bekeschus et al. (2020)	1	1	1	1	1	0	1	1	1	1	1	0	1	1	0	1	1	1	15
Cheng et al. (2017)	1	1	1	0	1	0	1	1	1	1	1	0	1	1	1	1	1	1	15
Dai et al. (2022)	1	1	1	1	1	1	1	1	1	1	1	0	1	1	1	1	1	1	17
Fadhil et al. (2024)	1	1	1	1	1	1	1	1	1	1	1	0	1	1	1	1	1	1	17
Jalili et al. (2016)	1	1	0	1	1	1	1	1	1	1	1	0	1	1	0	1	1	1	15
Jezeh et al. (2020)	1	1	1	1	1	1	1	1	1	1	1	0	1	1	0	1	1	1	16
Kim et al. (2020)	1	1	1	1	1	1	1	1	1	0	1	0	1	1	0	1	1	1	15
Kumar et al. (2014)	1	1	1	1	1	1	1	1	1	1	1	0	1	1	0	0	1	0	14
Liu et al. (2017)	1	1	1	1	1	1	1	1	1	1	1	0	1	1	0	1	1	1	16
Miron et al. (2023)	1	1	1	1	1	1	1	1	1	1	1	0	0	1	0	0	1	0	13
Miron et al. (2024)	1	1	1	1	1	1	1	1	1	1	1	1	1	1	1	1	1	1	18
Mirpour et al. (2014)	1	1	1	1	1	1	1	1	1	1	1	1	1	1	0	1	1	1	17

Misra et al. (2023)	1	1	1	1	1	1	1	1	1	1	1	0	1	1	0	1	1	1	16
Mokhtari et al. (2019)	1	1	1	1	1	1	1	1	1	1	1	0	1	1	0	1	1	1	16
Nagaya et al. (2019)	1	1	1	1	1	0	1	1	1	1	1	0	1	1	0	1	1	1	15
Nguyen et al. (2016)	1	1	1	1	1	1	1	1	1	1	1	0	1	1	0	1	1	1	16
Ninomiya et al. (2013)	1	1	1	1	1	1	1	1	1	1	1	0	1	1	1	0	1	1	16
Park et al. (2015)	1	1	0	1	1	1	1	1	1	1	1	0	1	1	0	0	1	1	14
Pranda et al. (2019)	1	1	1	1	1	1	1	1	1	1	1	0	1	1	0	1	1	0	15
Shakya et al. (2022)	1	1	1	1	1	0	1	1	1	1	1	0	1	1	1	1	1	1	16
Shen et al. (2025)	1	1	1	1	1	1	1	1	1	1	1	0	1	1	0	1	1	1	16
Subramanian et al. (2020)	1	1	1	1	1	0	1	1	1	1	1	0	1	1	0	1	1	0	14
Tanaka et al. (2024)	1	1	0	1	1	0	1	1	1	1	1	0	1	1	0	1	1	1	14
Terefinko et al. (2021)	1	1	1	1	1	1	1	1	1	1	1	0	1	1	0	1	1	1	16
Terefinko et al. (2024)	1	1	1	1	1	1	1	1	1	1	1	0	1	1	0	1	1	1	16
Wang et al. (2013)	1	1	1	1	1	1	1	1	1	1	1	0	1	1	0	1	1	1	16
Wang et al. (2020)	1	1	1	1	1	1	1	1	1	1	1	0	0	1	0	0	1	0	13
Wang et al. (2024)	1	1	1	1	1	1	1	1	1	1	1	0	1	1	0	1	1	0	15
Xiang et al. (2018)	1	1	1	1	1	1	0	1	1	1	1	0	1	1	1	1	1	1	16
Xu et al. (2018)	1	1	1	1	1	1	1	1	1	1	1	0	1	1	0	1	0	0	14
